# Effects of Weather Variables on Ascospore Discharge from *Fusarium graminearum* Perithecia

**DOI:** 10.1371/journal.pone.0138860

**Published:** 2015-09-24

**Authors:** Valentina Manstretta, Vittorio Rossi

**Affiliations:** Department of Sustainable Crop Production, Università Cattolica del Sacro Cuore, Piacenza, Italy; Seoul National University, REPUBLIC OF KOREA

## Abstract

*Fusarium graminearum* is a predominant component of the Fusarium head blight (FHB) complex of small grain cereals. Ascosporic infection plays a relevant role in the spread of the disease. A 3-year study was conducted on ascospore discharge. To separate the effect of weather on discharge from the effect of weather on the production and maturation of ascospores in perithecia, discharge was quantified with a volumetric spore sampler placed near maize stalk residues bearing perithecia with mature ascospores; the residues therefore served as a continuous source of ascospores. Ascospores were discharged from perithecia on 70% of 154 days. Rain (R) and vapor pressure deficit (VPD) were the variables that most affected ascospore discharge, with 84% of total discharges occurring on days with R≥0.2 mm or VPD≤11 hPa, and with 70% of total ascospore discharge peaks (≥ 30 ascospores/m^3^ air per day) occurring on days with R≥0.2 mm and VPD≤6.35 hPa. An ROC analysis using these criteria for R and VPD provided True Positive Proportion (TPP) = 0.84 and True Negative Proportion (TNP) = 0.63 for occurrence of ascospore discharge, and TPP = 0.70 and TNP = 0.89 for occurrence of peaks. Globally, 68 ascospores (2.5% of the total ascospores sampled) were trapped on the 17 days when no ascospores were erroneously predicted. When a discharge occurred, the numbers of *F*. *graminearum* ascospores sampled were predicted by a multiple regression model with R^2^ = 0.68. This model, which includes average and maximum temperature and VPD as predicting variables, slightly underestimated the real data and especially ascospore peaks. Numbers of ascospores in peaks were best predicted by wetness duration of the previous day, minimum temperature, and VPD, with R^2^ = 0.71. These results will help refine the epidemiological models used as decision aids in FHB management programs.

## Introduction

*Fusarium graminearum* Schwabe is often the prevalent species in the complex of organisms causing Fusarium head blight (FHB), an important disease affecting small-grain cereals [1, 2]. The fungus overwinters in crop debris where it produces asexual macroconidia and sexual ascospores, both of which cause disease on cereal heads in the following season [3]. Production of inoculum is favored by moist and warm conditions [4, 5]. Once produced, macroconidia are dispersed by splashing rain [6, 7], while ascospores are forcibly discharged from perithecia. Turgor pressure causes the ascus to stretch to the perithecium ostiole [8] and to eject ascospores at a distance from 4.0 to 4.6 mm [9, 10], which is sufficient to allow ascospores to become airborne in turbulent air currents [11]. In the laboratory, the entire life cycle takes about 2 weeks [12, 13, 14]; asci in perithecia mature and eject ascospores sequentially starting at 5 to 9 days after induction and continue to eject ascospores until 12 days after induction; maximal discharge occurs around day 6 [10, 12, 15, 16]. Complete discharge of a perithecium cohort was reported to require 6 h [12]. When ascospores become airborne, they traveled short distances in some reports [7, 17–23] and long distances in others [24–29].

Understanding how ascospores become airborne is important for disease control [30], and need for research on this topic was recently pointed out in a review by Keller et al. [31]. Studies on how meteorological factors affect the dynamics of airborne ascospores in the field as determined by spore samplers have had contradictory findings. Peaks of ascospore trapping were observed either during the night [19, 21, 27, 29, 32–34], during the day [35], or without diurnal pattern [26].

Some authors related observed diurnal patterns to meteorological conditions. Inch et al. [34] observed that ascospore trapping began in early afternoon, when temperature (T) was highest and relative humidity (RH) was lowest, but that trapping peaked at 21.00 h, when RH was highest. Paulitz & Seaman [36] reported that a decrease in T and the consequent rise in RH probably triggered ascospore discharge. Del Ponte et al. [33] reported peaks of ascospores on days with RH > 90%, and Paulitz [31] reported that ascospore trapping was inhibited on days with rain events, on cool days with RH constantly > 80%, or on days with high RH and intermittent rain.

Reported relationships between ascospore trapping and rain have also been inconsistent. Some authors found an increase in spore trapping on rainy days [34, 33, 37], while others found a reduction on rainy days [34, 35]. Some authors reported an increase in spore trapping between 1 to 7 days following rain, with a distinct lag phase between rainfall and ascospore release, suggesting that rainfall may be important for perithecial development and ascospore discharge [17, 19– 22, 34, 38, 39], but this pattern was not reported by others [20, 24, 27]. Peaks in ascospore trappings were also reported during non-rainy periods [40].

In all the previous field studies, it was impossible to separate the effects of meteorological conditions on ascospore production, discharge, and dispersal, and this may help explain the inconsistency. In other words, increases in airborne ascospores in the field could be due to environmental effects on ascospore production or transport from the original point of discharge rather than to effects on ascospore discharge.

Factors affecting ascospore discharge have also been studied in the laboratory. Ascospore discharge was initiated by an atmospheric saturation deficit [41], and discharge increased with RH and was maximum when RH exceeded 92% [12]. Discharge occurred between 10° and 30°C, with the optimum at 25°C [42]. Light did not significantly affect ascospore discharge, although discharge was slightly greater in light than in complete darkness [12]. These studies clarified the effect of single, constant weather factors on discharge but they did not indicate how discharge is affected by interacting factors under natural, fluctuating conditions.

The aim of the present work was to separate the effect of weather on the discharge of *F*. *graminearum* ascospores from the effect of weather on the production and maturation of ascospores in perithecia. This was accomplished by quantifying the discharge of ascospores from maize stalk residues bearing perithecia with mature ascospores. The residues therefore served as a continuous source of ascospores, i.e., ascospore discharge did not depend on perithecia maturing during the study.

## Materials and Methods

### Production of perithecia on maize stalk residues

Overwintered maize stalks were collected from commercial fields at the end of winter (February) in 2012, 2013, and 2014. The owner of the land gave permission to collect maize residues on this site. Maize stalks were cut into 15-cm lengths and were soaked in water overnight and then autoclaved twice with a 24 h interval between the two sterilization cycles (120°C for 20 min each cycle). The maize stalk pieces were then submerged in a spore suspension (2×10^4^ spores/mL water) for 2 min. The suspension was obtained by mixing equal amounts of spore suspensions of two *F*. *graminearum* strains. The strains used had previously been tested for their ability to produce perithecia and were: i) a strain isolated from wheat in Italy (Mycological collection Università Cattolica del Sacro Cuore), and ii) a strain isolated from oats in Germany (Istitut fur landwirtschaftliche Kulturen, Bundesanstalt fur Zuchtungsforschung an Kulturpflanzen). Twelve inoculated stalks were incubated for 1 week at room temperature (20° to 25°C) in a saturated atmosphere to allow colonization and were then placed over a clay soil in a plastic box (20 × 25 × 10 cm) with holes in the bottom to allow water to drain during rainfall. The plastic box was placed outdoors on a grass lawn and was kept constantly wet until perithecia were produced. Perithecium maturation was checked by randomly picking 50 perithecia with a needle, crushing them on a microscope slide, and observing the resulting material with a microscope (200× magnification); the perithecia were considered mature when they contained fully formed, septate ascospores. Before ascospore sampling was started, most of the perithecia were mature.

### Ascospore sampling

The plastic box containing maize stalks with mature perithecia (see previous section) was placed on a pedestal that was fastened to the upper part of a 7-day recording volumetric spore sampler (Lanzoni VPPS 2000, Bologna, Italy). The box was positioned so that the stalks were in front of the air aspiration orifice, at a distance between 15 and 40 cm, depending on the location of the stalks in the box.

The spore sampler was installed outdoors on the campus of the University of Piacenza (North Italy) on 16 April 2012, 23 April 2013, and 5 May 2014. The spore sampler was operated using a 220 V 50 Hz power source and was adjusted to sample air at 10 L/min at 115 cm above the ground. The tape that served as the trapping surface was 14 mm wide and was coated with a thin layer of silicon and glycerol (Lanzoni, Bologna, Italy); it was removed weekly and cut for microscopic examination. The tape was examined microscopically (250× magnification) by scanning four equidistant transects across the long axis of the tape at intervals of 2 mm (equivalent to 1 h of trapping). The number of *F*. *graminearum* ascospores observed in each 1-h transect was corrected for the proportion of the tape examined and the volume of air sampled: it was then expressed as number of spores trapped per m^3^ of air per h or per day. The spore counts were not adjusted for trap efficiency.

During spore sampling periods, stalk pieces were examined twice each week with a dissecting microscope (40× magnification), and 10 random perithecia were collected with a needle. The collected perithecia were crushed on a microscope slide and examined with a microscope (200× magnification) to verify the presence of fully formed, septate ascospores. If no ascospores were found, the experiment was stopped; it occurred on 11 May 2012, 6 July 2013, 26 June 2014.

Air temperature (T, °C), relative humidity (RH, %), total rainfall (R, mm), and wetness duration (WD, min) were recorded hourly by an automatic weather station (MeteoSense 2.0, Netsens s.r.l., Firenze, Italy) installed at the experimental site during the whole period when the spore sampler operated. Vapor pressure deficit (in hPa) was calculated using T and RH data following Buck [43], as follows: VPD = (1-RH/100)×(6.11×exp((17.47×T)/(239+T))).

### Data analysis

Hourly weather data were used to calculate the following variables on a daily time scale (a day began at 01.00 h and ended at 24.00 h): average, maximum, and minimum T (Tav, Tmin, and Tmax, respectively); average, maximum, and minimum RH (RHav, RHmin, and RHmax, respectively); total R (Rt); total WD (WDt, in hours); number of hours with RH ≥ 90% (RH90); and average vapor pressure deficit (VPD).

The relationships between the weather variables listed in the previous paragraph and the presence of *F*. *graminearum* ascospores (i.e., ≥ 1 ascospore/m^3^ air), or a peak of ascospores (i.e., ≥ 30 ascospores/m^3^ air [19]) on a day were investigated with three analyses. In the first analysis, each i^th^ day was classified as “0” if no ascospores were sampled (or no peaks were found) or as “1” if ascospores (or peaks) were found (regardless of the number ascospores found). Differences in the weather variables between the two groups (days with or without ascospores, or peaks) were determined using a *t*-test. In the second analysis, the Pearson’s correlation coefficients were calculated between the natural logarithm of ascospores sampled on each day i and the weather variables on day i and i-1. The natural logarithm was calculated as: ln(x+1), where x = ascospores/m^3^ air per day to make variances homogeneous. In the third analysis, the weather variables of day i or i-1 were considered as possible predictors of the presence (or peaks) of ascospores in a receiver operating characteristic (ROC) analysis [44]. The ROC curve was plotted as the true positive proportion (TPP, or sensitivity) of the prediction as a function of the false positive proportion (FPP, or 1-specificity) for different cut-off points of each weather variable. For instance, the cut-off points for RH90 were: 0 h (i.e., no hours with RH ≥ 90% in the day), 1 (1 hour with RH ≥ 90% in the day), 2, 3, and so on up to 24 hours with RH ≥ 90% in the day. Each point on the ROC curve represents a sensitivity/specificity pair corresponding to a particular cut-off point; the closer the ROC curve is to the upper left corner of the plot, the higher the overall accuracy of the test, i.e., the ratio between the number of cases assigned to the correct class and the number of cases that actually belong to that class [45]. For every possible cut-off point, the proportion of days correctly classified as positive (TPP = True Positive Proportion) or negative (TNP = True Negative Proportion), or wrongly classified as negative (FNP = False Negative Proportion) or positive (FPP = False Positive Proportion) was calculated. The area under the ROC curve (AUROC) and its standard error were calculated to measure how well a weather variable distinguished between the two groups (presence / absence of ascospores or peaks). The AUROC lies in the interval between 0.5 and 1.0, and a larger area indicates better performance. The *P*-value was calculated as the probability that the AUROC was different from the null hypothesis, i.e., that AUROC = 0.5 (the ROC curve coincided with the diagonal) and that the variable under study did not distinguish between the two groups.

The relationships between the weather variables and the natural logarithm of numbers of *F*. *graminearum* ascospores on any sampling day, or on days with ascospore peaks, were investigated with a multiple regression analysis, with n = 154 sampling days or n = 27 ascospore peak days. The weather variables calculated for day i and i-1 were regressed against the natural logarithm of ascospore numbers on day i. Dycotomic variables accounting for the year of sampling, the presence or absence of ascospores on day i and i-1, and for the cut-off points from the ROC analysis—e.g., R02 (i.e., rain ≥ 0.2 mm) and VPD11 (i.e., VPD ≥ 11 hPa)—were also considered in the regression analysis. The best regression model was selected through a stepwise forward selection procedure that involved starting with no variables in the model, testing the addition of each variable using the probability of F as comparison criterion (with *P* = 0.05 for entering into or exiting from the model), adding the variable (if any) that most improves the model, and repeating this process until the model was no longer improved. Once the best model was selected, residuals were calculated as observed—predicted values, and their dimension and distribution were analysed.

PASW Statistic 18 (IBM, Armonk, NY) was used for all statistical analyses.

## Results

**Fig 1:**
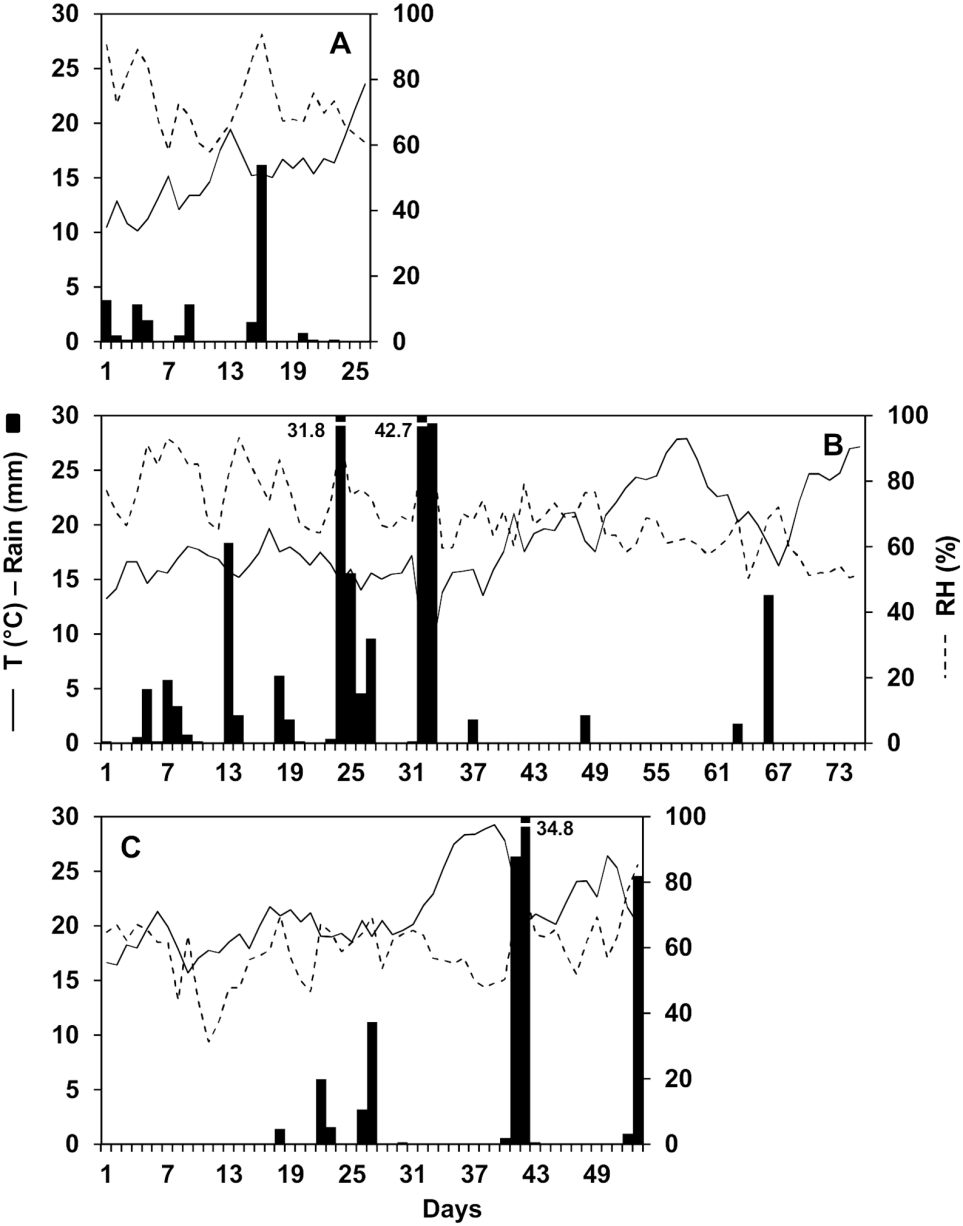
Weather data during the sampling periods. Air temperature (T), relative humidity (RH), and rainfall at the University of Piacenza (North Italy) during the periods in 2012 (A), 2013 (B), and 2014 (C) when *Fusarium graminearum* ascospores were sampled from the air above maize stalk residues bearing mature perithecia.

The period in which there were mature perithecia on maize stalk residues was 26 days long in 2012, 75 days in 2013, and 53 days in 2014. Weather data measured during these three sampling periods are shown in Fig 1. In 2012, the overall average daily temperature was 15.3°C, with a minimum (min) of 10.1°C and a maximum (max) of 23.6°C; the average daily RH was 72.3%, with a min of 58.0% and a max of 93.8%. Total rainfall was 33.2 mm on 12 rainy days (representing 48% of the total number of days, most rainy days had < 2 mm of rain). In 2013, the average daily temperature during the experiment was higher than in 2012 (18.8°C, min 8.7°C, and max 27.7°C) and RH was lower (69.8%, min 50.3% and max 93.5%); total rainfall was about six-times higher (total of 200.2 mm on 25 rainy days). Several consecutives days without rain (i.e., dry periods) were recorded between the end of May and June in 2013. In 2014, the experiment began later than in the previous years, and the average daily temperature was higher (21.2°C, min 15.7°C, and max 29.2°C) and RH was lower (59.3%, min 31.2%, and max 85.3%); total rainfall was 111.2 mm on 12 rainy days. Dry periods were recorded from day 1 to 21, from day 31 to 39, and from day 44 to 51. Average rain amount per rain event was higher in 2013 and 2014 than in 2012 (8.0, 9.2, and 2.8 mm, respectively). Two rain events with > 30 mm were recorded in 2013 and one in 2014; in 2012, the highest rain amount in 1 day was 16.2 mm.

**Fig 2:**
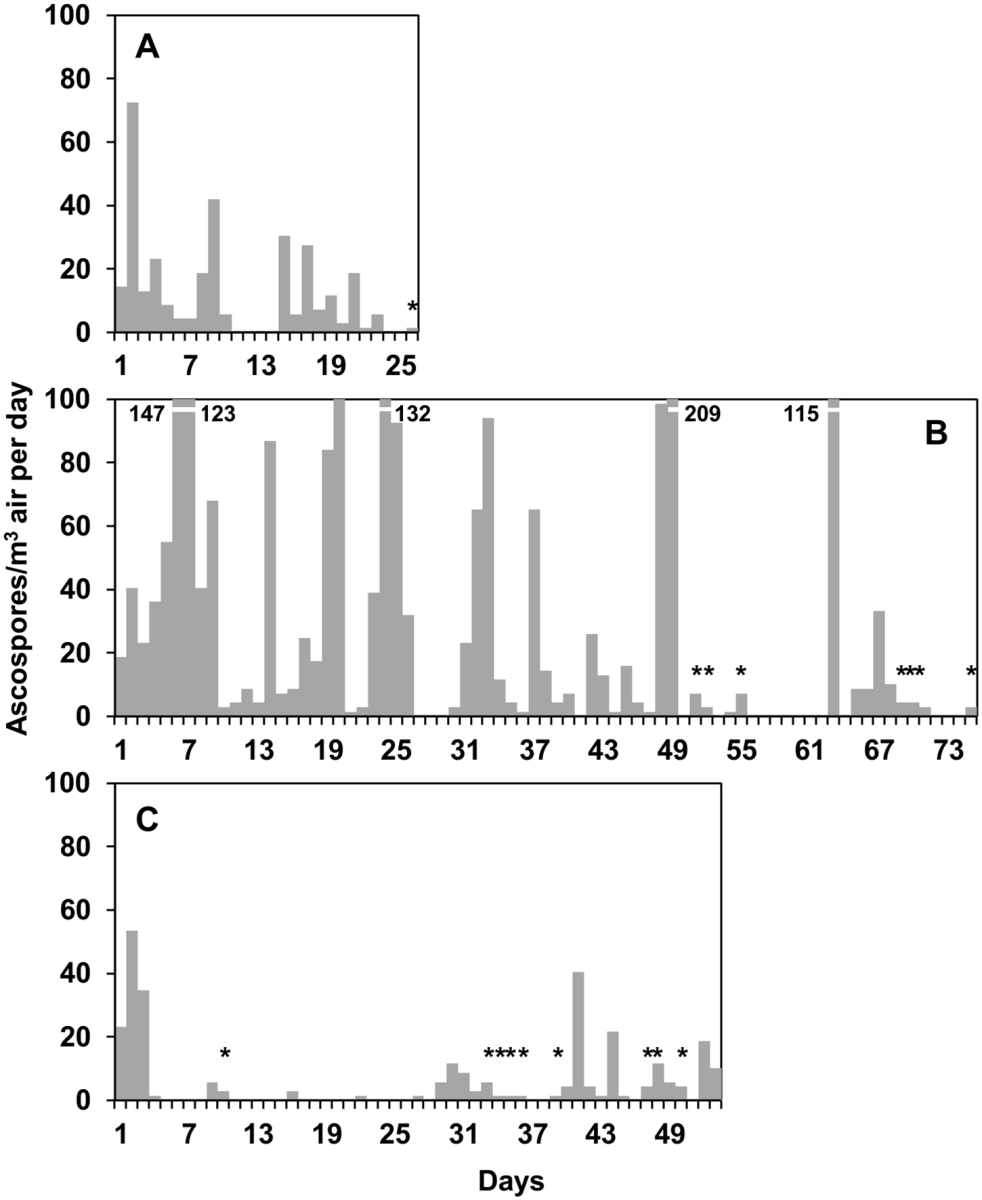
Numbers of *Fusarium graminearum* ascospores sampled daily. Ascospores were sampled from the air above maize stalk residues bearing mature perithecia at the University of Piacenza (North Italy) in 2012 (A), 2013 (B), and 2014 (C). * indicates days in which no ascospores were erroneously predicted according to the discharge criteria R ≥ 2 mm/day or VPD ≤ 11 hPa.

Ascospores were trapped on 108 of the 154 days (70% of the total days); numbers of ascospores ranged from 2 to 209 ascospores/m^3^ air per day (Fig 2). There were 27 peaks of ascospores (i.e., days with ≥ 30 ascospores/m^3^ air); in aggregate, 2,039 ascospores (76% of the total ascospores trapped) were trapped during these peaks.

Weather was significantly cooler and moister on days with ascospores than on days without ascospores (Table 1). Across all three periods, Tav and Tmax were 3.0 and 4.3°C lower on days with ascospores than on days without ascospores; RHav was 12.1% higher and VPD was 4.6 hPa lower on days with ascospores than on days without ascospores. On days without ascospores, WDt and RH90 were < 1 h, while on days with ascospores, WDt = 5 h and RH90 = 4 h. Similarly, days with ascospore peaks were significantly cooler and moister than days without peaks (Table 1); Tmax was 3.6°C lower, RHmin was 10.4% higher, and VPD was 3.2 hPa lower on days with peaks than on days without peaks (Table 1).

**Table 1:** Weather data during periods with and without discharge of *Fusarium graminearum* ascospores, and with and without peaks of discharge, in North Italy in 2012 to 2014.

		Ascospore discharge^3^ (Re)			Ascospore peak^4^ (Pe)			
Weather Variable^1^	Group^2^	Average	s.e.	*P*(0,1)^5^	Average	s.e.	*P*(0,1)^5^	*P*(Re,Pe)^6^
**Tav**	0	21.2	0.54	<0.001	19.7	0.38	<0.001	<0.001
	1	18.2	0.41		15.9	0.56		
**Tmin**	0	14.0	0.55	0.046	13.4	0.34	0.027	0.129
	1	12.7	0.35		11.9	0.52		
**Tmax**	0	28.2	0.61	<0.001	26.2	0.45	<0.001	<0.001
	1	23.9	0.52		20.3	0.84		
**RHav**	0	58.1	1.29	<0.001	64.1	0.95	<0.001	<0.001
	1	70.2	1.08		78.4	1.88		
**RHmin**	0	36.7	1.34	<0.001	42.6	1.07	<0.001	<0.001
	1	49.4	1.41		59.8	3.15		
**RHmax**	0	81.1	1.37	<0.001	85.4	0.76	<0.001	<0.001
	1	89.4	0.68		93.9	0.84		
**WDt**	0	0.9	0.31	<0.001	2.6	0.37	<0.001	0.039
	1	5.0	0.52		9.2	1.03		
**RH90**	0	0.7	0.27	<0.001	1.9	0.33	<0.001	<0.001
	1	4.0	0.54		8.0	1.33		
**VPD**	0	12.5	0.59	<0.001	10.2	0.43	<0.001	0.001
	1	7.9	0.46		4.7	0.52		
**Rt**	0	0.3	0.22	0.022	1.3	0.41	0.021	<0.001
	1	3.0	0.76		6.8	2.24		

^1^ Tav, Tmin, Tmax = average, min, and max daily temperature; RHav, RHmin, RHmax = average, min, and max daily relative humidity; WDt = total wetness duration; RH90 = hours with RH > 90%; VPD = vapor pressure deficit; Rt = total rain.

^2^ 0 = days with no ascospore discharge or no ascospore peaks; 1 = days with ascospore discharge or ascospore peak.

^3^ Ascospores were sampled with a volumetric spore sampler from the air above maize stalk residues bearing mature *F*. *graminearum* perithecia.

^4^ Peaks are defined as days with ≥ 30 ascospores / m^3^ air.

^5^ Probability level of the *t*-test for differences between the two ascospore groups (0 = no, 1 = yes).

^6^ Probability level of the *t*-test of differences between presence and peak of ascospores.

The presence of ascospores was associated with rain: ascospores were detected on 45 of the 49 rainy days (i.e., days on which R ≥ 0.2 mm), and 42 of the 46 days without ascospores were dry; however, 63 of the 108 days with ascospores were dry (Fig 2). The use of R ≥ 0.2 mm/day as a cutoff point to predict ascospore presence gave a TNP (specificity) of 0.91 but a TPP (sensitivity) of 0.42, which is a posterior probability that ascospores were trapped when not predicted Prob(P-/O+) = 0.58 (Table 2). The overall accuracy was = 0.56. The ROC analysis showed that AUROC = 0.336 ± 0.044 (*P* = 0.001) (Table 3); increasing the rain quantity of the cutoff did not increase the prediction accuracy (not shown). The use of rain as predictor of ascospore peaks gave AUROC = 0.184 ± 0.048 (*P* = 0.04) (Table 3). Twenty-two of the 27 ascospore peaks occurred on rainy days, but no peaks were detected on 27 of the 49 rainy days (Fig 2). Therefore, when rain was used as a predictor of ascospore peaks, the posterior probability that ascospore peaks did not occur when predicted Prob(P+/O-) = 0.55 (Table 2).

**Table 2:** Comparison between observed and predicted discharges and peaks of *Fusarium graminearum* ascospores in North Italy in 2012 to 2014. Predictions were based on rain alone or rain and vapor pressure deficit.

		Proportion^3^				Overall accuracy	Likelyhood ratio		Prior probability		Posterior probability			
Ascospore^1^	Predicting variable^2^	TPP	FNP	FPP	TNP		LR(+)	LR(-)	O+	O-	P+/O+	P-/O-	P+/O-	P-/O+
**Discharge**	Rain	0.42	0.58	0.09	0.91	0.56	4.79	0.639	0.70	0.30	0.92	0.42	0.08	0.58
**Peak** ^**4**^	Rain	0.81	0.19	0.21	0.79	0.79	3.83	0.235	0.18	0.82	0.45	0.96	0.55	0.04
**Discharge**	Rain, VPD	0.84	0.16	0.37	0.63	0.78	2.28	0.250	0.70	0.30	0.84	0.73	0.16	0.27
**Peak**	Rain, VPD	0.70	0.30	0.11	0.89	0.86	6.38	0.333	0.18	0.82	0.58	0.94	0.42	0.06

^1^ Ascospores were sampled with a volumetric spore sampler from the air above maize stalk residues bearing mature *F*. *graminearum* perithecia.

^2^ Rain ≥ 0.2 mm/day; vapor pressure deficit VPD ≤ 11 hPa for discharges; VPD ≤ 6.35 hPa for peaks.

^3^ TPP = True Positive Proportion (sensitivity); FNP = False Negative Proportion; FPP = False Positive Proportion; TNP = True Negative Proportion (specificity)

^4^ Peaks are defined as days with ≥ 30 ascospores / m^3^ air.

**Table 3:** Characteristics of the ROC curves obtained using weather data as predictors of discharge and peaks of *Fusarium graminearum* ascospores in North Italy in 2012 to 2014.

	Ascospore discharge^2^		Ascospore peak^3^	
Predicting variable^1^	AUROC^4^	s.e.	AUROC	s.e.
**Tav**	0.717	0.043	0.774	0.043
**Tmin**	0.591	0.049	0.615	0.052
**Tmax**	0.748	0.041	0.823	0.042
**RHav**	0.190	0.035	0.149	0.033
**RHmin**	0.233	0.038	0.184	0.041
**RHmax**	0.228	0.041	0.172	0.041
**RH90**	0.307	0.043	0.191	0.047
**WDt**	0.225	0.038	0.129	0.031
**VPD**	0.799	0.036	0.849	0.036
**Rt**	0.336	0.044	0.184	0.048

^1^ Tav, Tmin, Tmax = average, min, and max daily temperature; RHav, RHmin, RHmax = average, min, and max daily relative humidity; WDt = total wetness duration; RH90 = hours with RH > 90%; VPD = vapor pressure deficit; Rt = total rain.

^2^ Ascospores were sampled with a volumetric spore sampler from the air above maize stalk residues bearing mature *F*. *graminearum* perithecia.

^3^ Peaks are defined as days with ≥ 30 ascospores / m^3^ air.

^4^ Area under the ROC curve.

**Fig 3:**
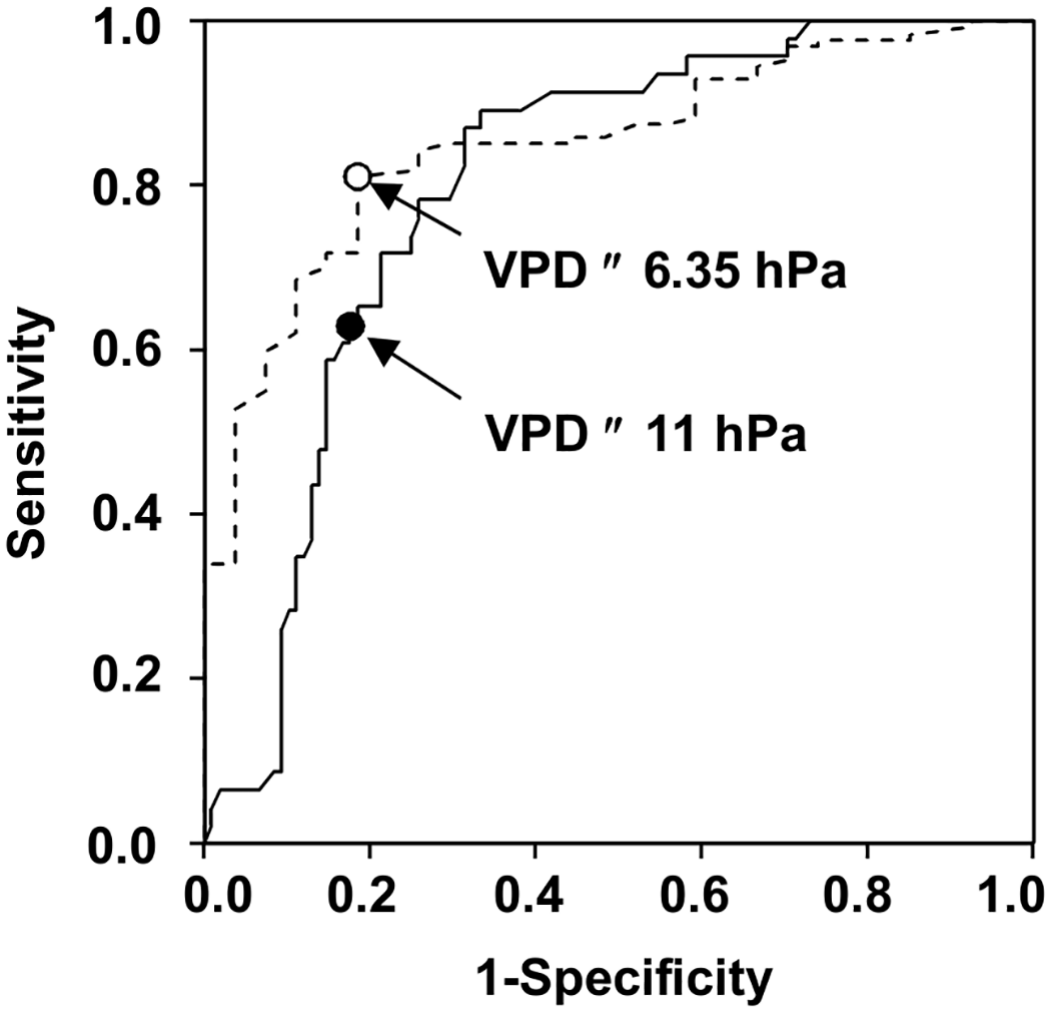
ROC curve. Sensitivity vs. 1-Specificity (ROC curve) in predicting discharge (line) and peaks (dotted line) of *Fusarium graminearum* ascospores as affected by different cut-off points for the number of hours per day with vapor pressure deficit (hPa) ≤ the cut-off point at the University of Piacenza (North Italy) in 2012 to 2014. Points and numbers inside the plot are the best cut-off points.

The ROC analysis showed that VPD was the best meteorological variable for predicting both ascospore trapping (AUROC = 0.799 ± 0.036; *P* < 0.001) and ascospore peaks (AUROC = 0.849 ± 0.036; *P* < 0.001) (Table 2); the best cut-off points for the two ROC curves were VPD ≤ 11 and ≤ 6.35 hPa, respectively (Fig 3). Maximum and average daily temperature resulted in AUROC values > 0.7, while use of RH, WD, or numbers of hours with RH > 90% resulted in AUROC values < 0.3 (Table 3).

When the criteria R ≥ 0.2 mm/day and VPD ≤ 11 hPa were combined to predict ascospore presence, the overall accuracy of the prediction increased from 0.56 (prediction based on rain only) to 0.78, and the sensitivity was TPP = 0.84 and specificity TNP = 0.63 (Table 2). The posterior probability that there were ascospores when predicted was Prob(P+/O+) = 0.84, and the probability that there were no ascospores when not predicted was Prob(P-/O-) = 0.73. The probability that there were ascospores when not predicted Prob(P-/O+), dropped from 0.56 (prediction based on rain only) to 0.29 (prediction based on rain and VPD) (Table 2). The 17 days when no ascospores were erroneously predicted by the use of rain and VPD as predictors are shown in Fig 2. Globally, 68 ascospores were trapped on these days, which accounted for 2.5% of the total ascospores trapped, with a maximum of 12 ascospores/m^3^ air on 21 June 2014 (Fig 2C).

When the criteria R ≥ 0.2 mm/day and VPD ≤ 6.35 hPa were combined, the overall accuracy of ascospore peak prediction increased from 0.78 (prediction based on rain only) to 0.86; sensitivity and specificity were TPP = 0.70 and TNP = 0.89, respectively (Table 2). The posterior probability that there was a peak when predicted was lower than the probability that there was not a peak when not predicted, with Prob(P+/O+) = 0.58 (which was 0.45 for predictions based on rain only) and Prob(P-/O-) = 0.94 (Table 2). Therefore, the above criteria were more accurate for predicting days with no ascospore peaks than days with peaks.

**Fig 4:**
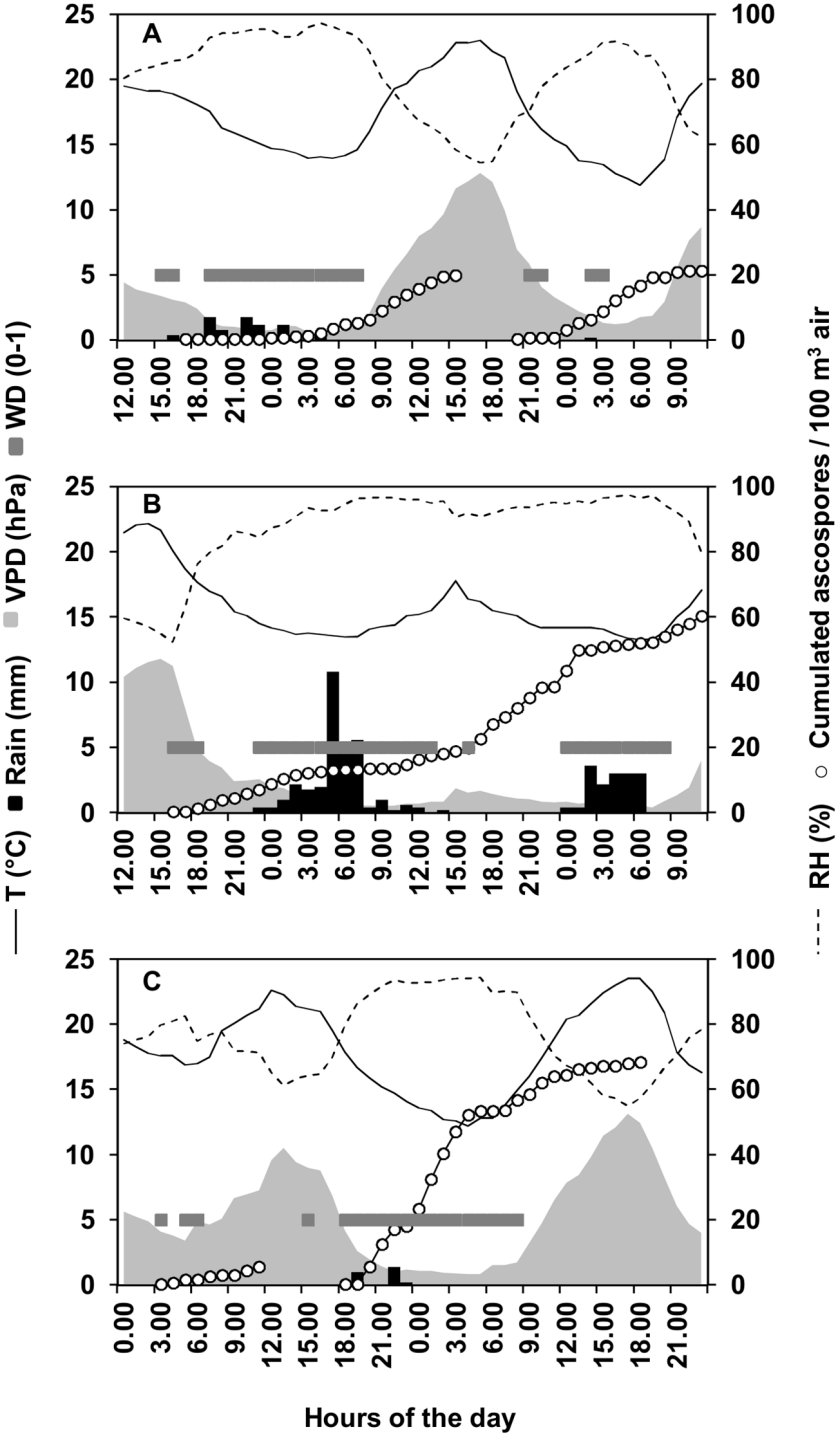
Weather data during sampling and number of ascospores sampled in specific periods. Hourly air temperature (T), relative humidity (RH), rain, wetness duration (WD), vapor pressure deficit (VPD), and numbers of *Fusarium graminearum* ascospores sampled from the air above maize stalk residues bearing mature perithecia at the University of Piacenza (North Italy) in 2013. Panels show three different period of 48 hours: from 12.00 of 10 May to 11.00 of 12 May (A), from 12.00 of 15 May to 11.00 of 16 May (B) and from 00.00 of 9 June to 23.00 of 10 June (C).

A relationship between ascospore discharge and rain and VPD and was also indicated by analysis of the hourly data. For instance, on 10 May 2013 (Fig 4A), ascospore discharge was triggered by a 10-h rain period (8.2 mm rain in total) with prolonged wetness and low VPD. The discharge continued (with a higher ascospore discharge rate) after the rain had ended and VPD increased, and the discharge stopped 23 h after starting, when VPD = 12.2 hPa. The ascospore discharge restarted after a 4-h interruption, when VPD dropped to 6.9 hPa. On 15 May 2013 (Fig 4B), ascospore discharge began at 16.00, when VPD rapidly dropped from 12.0 to 8.1 hPa. This discharge continued for 44 h with low VPD and two distinct 16-h-long and 4-h-long rain periods (32.2 and 6.6 mm rain, respectively). Similarly, on 8 June 2013 (Fig 4C), ascospore discharge began when VPD dropped from 8.0 to 4.1 hPa, stopped as VPD increased, and restarted when VPD dropped again to 4.2 hPa, 1 h before 1 mm of rain fell. On the following day, ascospore discharge continued at high rate while VPD remained low, at a lower rate as VPD increased, and finally stopped when VPD = 12.4 hPa.

Ascospore numbers were significantly (*P* ≤ 0.001) correlated with weather variables. Correlation coefficients were high and positive for RH (r = 0.64) and WD (r = 0.61), while they were negative for Tmax (r = -0.56) and VPD (r = -0.605); there was a weak, positive correlation with rainfall (r = 0.30) (Table 4). Similar correlations were found for the day before ascospores were trapped (i.e., day i-1), but the correlation coefficients were lower for day i-1 than for day i (Table 4).

**Table 4:** Correlation coefficients between daily numbers of airborne *Fusarium graminearum* ascospores and weather data registered during the day of ascospore trapping (Day i) and the day before trapping (Day i-1) in North Italy in 2012 to 2014.

	Day i		Day i-1	
Weather variable^1^	r^2^	*P*	r	*P*
**Tav**	-0.491	<0.001	-0.346	<0.001
**Tmin**	-0.258	0.001	-0.150	0.064
**Tmax**	-0.559	<0.001	-0.419	<0.001
**RHav**	0.640	<0.001	0.493	<0.001
**RHmin**	0.596	<0.001	0.490	<0.001
**RHmax**	0.545	<0.001	0.371	<0.001
**WDt**	0.610	<0.001	0.423	<0.001
**RH90**	0.532	<0.001	0.372	<0.001
**VPD**	-0.605	<0.001	-0.442	<0.001
**Rt**	0.299	<0.001	0.234	0.004

^1^ Tav, Tmin, Tmax = average, min, and max daily temperature; RHav, RHmin, RHmax = average, min, and max daily relative humidity; WDt = total wetness duration; RH90 = hours with RH > 90%; VPD = vapor pressure deficit; Rt = total rain.

^2^ Pearson correlation coefficient (n = 154); ascospores were sampled with a volumetric spore sampler from the air above maize stalk residues bearing mature *F*. *graminearum* perithecia; ascospore numbers were transformed by the function ln(x+1) for the analysis.

**Fig 5:**
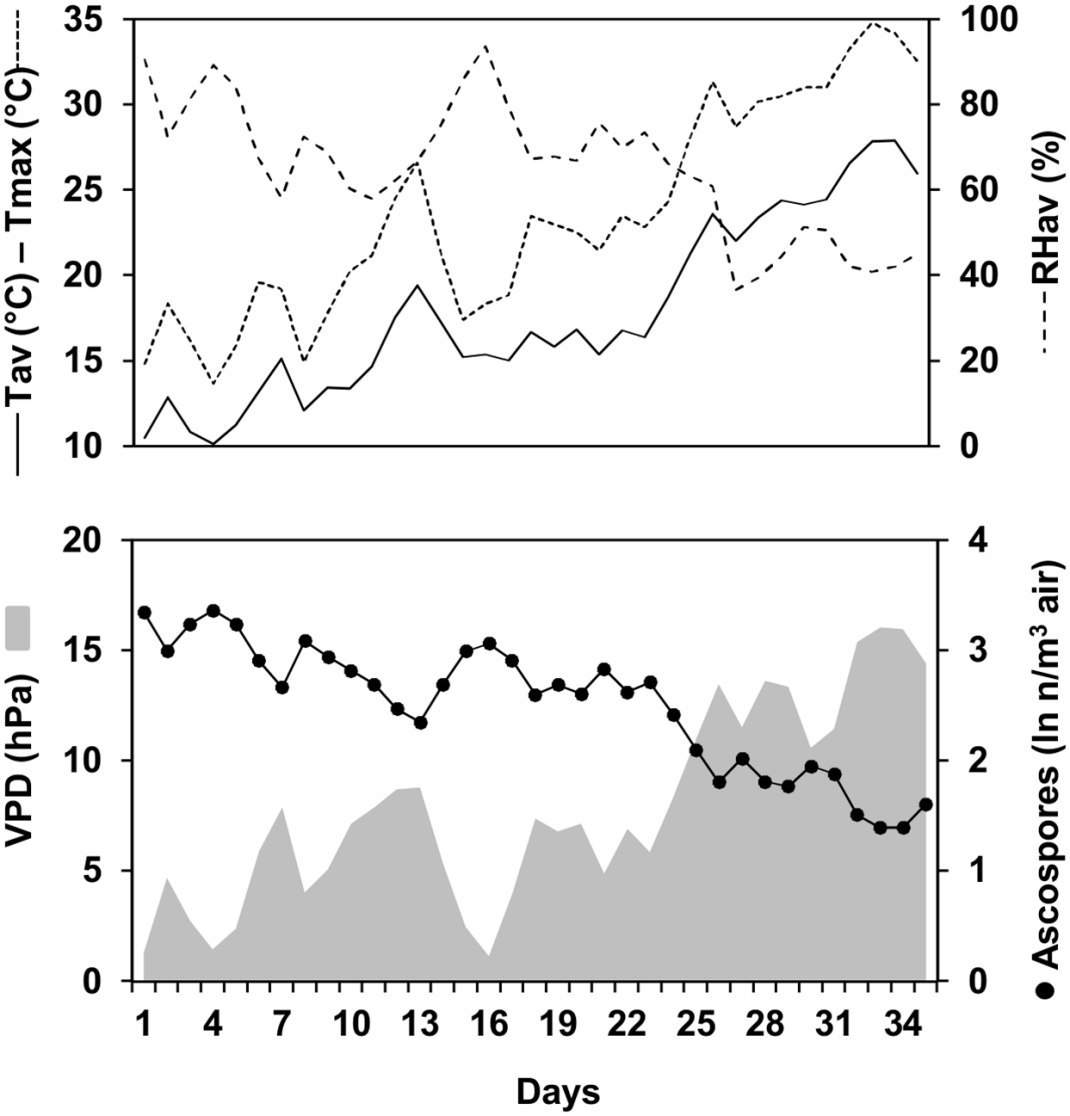
Model prediction. Numbers of *Fusarium graminearum* ascospores predicted by model (1) (see Table 5) based on average air temperature (Tav), maximum air temperature (Tmax), and vapor pressure deficit (VPD). Average relative humidity (RHav) is shown as a component for calculating VPD. Predicted ascospore numbers are expressed as ln(x+1).

Based on a stepwise regression procedure, the following variables were selected as being most useful for predicting the number of ascospores sampled (on a ln scale): T_i_, Tmax_i_, and VPD_i_. Based on regression model (1) (Table 5), on any day with ascospores (accounted for by the dichotomic variable ASC_i_), the ascospore number decreased as temperature and VPD of the day increased. Fig 5 shows the numbers of ascospores predicted by the model along with temperature (as both T and Tmax), RH, and VPD on a set of selected days during which temperature increased. Note that the trend in ascospore numbers was opposite to the trends in both temperature and VPD.

**Table 5:** Parameters and statistics of the regression models describing the relationships between weather data and daily numbers of airborne *Fusarium graminearum* ascospores sampled in North Italy in 2012 to 2014.

Data set^1^	Weather variables^2^		Parameters^3^		s.e.^4^	*P*	R^2^	s.e.^5^
**All days—Model (1)**	X_0_	Intercept	β_0_	2.093	0.552	<0.001	0.682	0.87
	X_1_	Asc_i_(0,1)	β_1_	1.977	0.171	<0.001		
	X_2_	Tav_i_	β_2_	0.181	0.067	0.008		
	X_3_	Tmax_i_	β_3_	-0.184	0.063	0.004		
	X_4_	VPD_i_	β_4_	-0.061	0.016	0.027		
**Peak days—Model (2)**	X_0_	Intercept	β_0_	3.474	0.304	<0.001	0.710	0.29
	X_1_	Tmin_i_	β_1_	0.040	0.021	0.044		
	X_2_	VPD_i_	β_2_	-0.05	0.021	0.026		
	X_3_	WDt_i-1_	β_3_	0.063	0.012	<0.001		

^1^ In the first data set, all sampling days were considered, whether or not ascospores were discharged from perithecia (n = 154); in the second data set, only days with ascospore peaks, i.e., ≥ 30 ascospores / m^3^ air per day, were considered (n = 27).

^2^ Tav, Tmin, Tmax = average, min, and max daily temperature; WDt = total wetness duration; VPD = vapor pressure deficit; Asc(0,1) = dycotomic variables with 0 and 1 being no or yes ascospores, respectively.

^3^ Parameters of the following regression model: Y = β_0_ + β_1_X_1_ + … + β_n_X_n_; Y = natural logarithm of ascospore numbers. Ascospores were sampled with a volumetric spore sampler from the air above maize stalk residues bearing mature *F*. *graminearum* perithecia; ascospore numbers were transformed by using the function ln(x+1) for the analysis.

^4^ Standard error of the parameters.

^5^ Standard error of the estimates.

**Fig 6:**
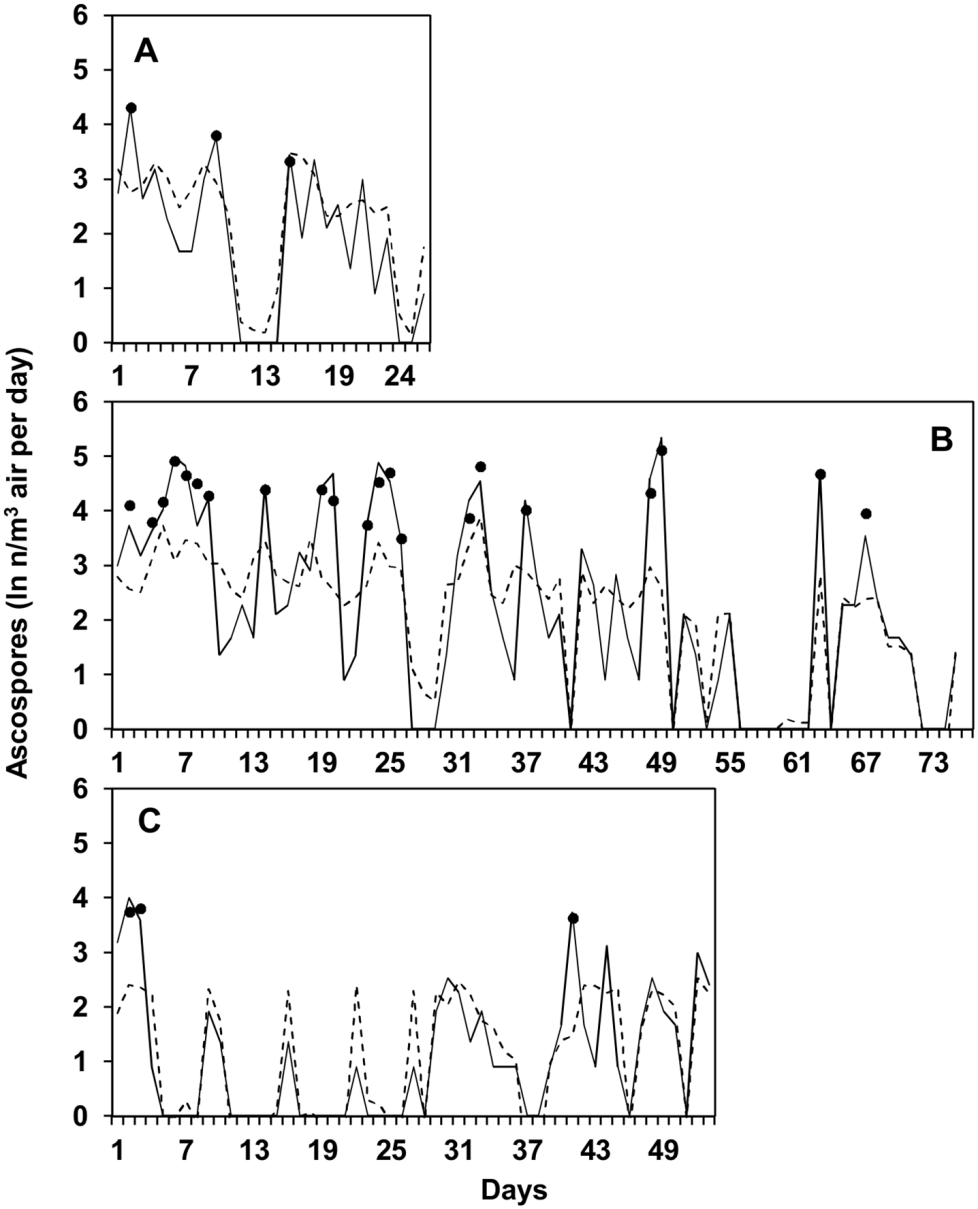
Model prediction in the sampling periods. Numbers of *Fusarium graminearum* ascospores predicted daily by model (1) (dotted line) and model (2) (points) (see Table 5) and observed (line) at the University of Piacenza (North Italy) in 2012 (A), 2013 (B), and 2014 (C). Ascospores were sampled with a volumetric spore sampler from the air above maize stalk residues bearing mature perithecia; ascospore numbers are expressed as ln(x+1).

**Fig 7:**
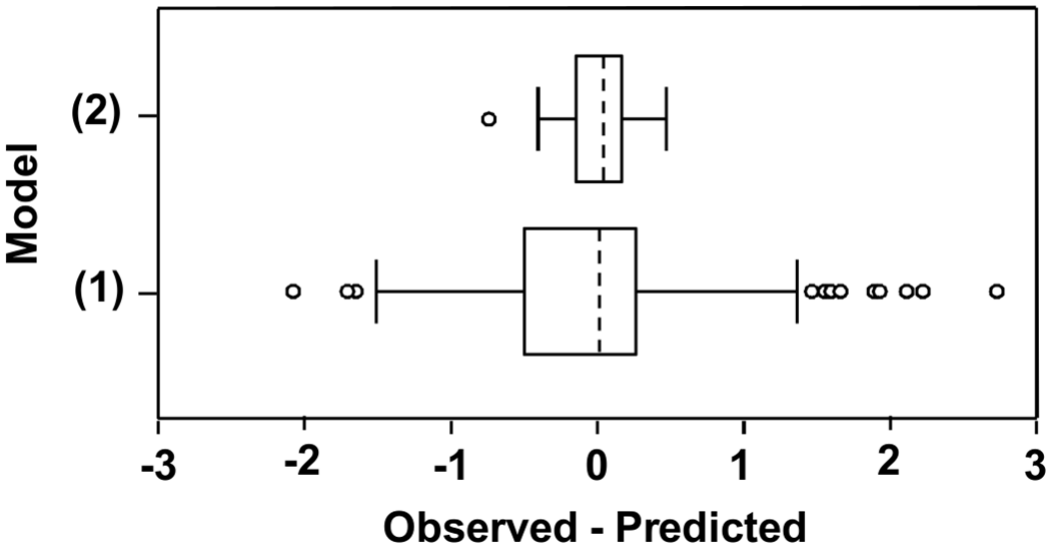
Box-plot of the residuals. Box-plot of the residuals (observed—predicted) for numbers of *Fusarium graminearum* ascospores predicted daily by model (1) and model (2) (see Table 5).

Model (1) described actual ascospore trappings with R^2^ = 0.68 (Fig 6). Most of the residuals observed—predicted were in the interval ± 1.5, with a positive kurtosis (= 0.81, meaning that residual distribution is peaked relative to a normal distribution) and some outliers extending to the minimum of -2.11 and the maximum of 2.76 (Fig 7). The distribution of residuals had a small positive asymmetry (skewness = 0.48 ± 0.19), meaning that model (1) underestimated the real data and especially ascospore peaks (Fig 6); the average of residual was -0.04 ± 0.07 for all days and 1.26 ± 0.12 for the peak days.

Ascospore peaks were best predicted by WD_i-1_, Tmin_i_, and VPD_i_, as in equation (2) (Table 5). Based on regression model (2), ascospore numbers in peaks depended on the humidity of the previous day (specifically, duration of the wet period, WD) in addition to temperature (specifically Tmin) and VPD of the peak day. Model (2) described the actual numbers of ascospores in peaks with R^2^ = 0.71 (Fig 6). All of the residuals observed—predicted were in the interval ± 0.5, with one outlier (Fig 7).

## Discussion

To our knowledge, this is the first published paper investigating the effect of natural weather conditions on the discharge of *F*. *graminearum* ascospores. To separate ascospore discharge from ascospore production and maturation, ascospores were sampled from the air just above maize stalk residues bearing mature perithecia, i.e., the mature perithecia on the residues served as a continuous source of ascospores. Previous papers established relationships between weather variables and numbers of airborne ascospores in the field [19, 21, 27, 33, 34, 37, 40, 46, 47]. In these papers, however, the dynamics of ascospore trapping were the result of weather conditions on multiple mechanisms, i.e., on ascospore maturation, discharge, and transport from the original point of discharge. Because the inoculum status was unknown and was probably highly variable, it is not surprising that the results of these previous field studies were often inconsistent. In other papers, the effects of humidity and temperature were investigated under controlled conditions [12, 41, 42]. These papers described how the dispersal of ascospores from *F*. *graminearum* is affected by single weather variables, but they did not account for the interaction between these variables and for their daily fluctuations. Maldonado-Ramirez & Bergstrom [48] conducted an experiment similar to the one described in the present study in order to document and characterize temporal patterns of ascospore discharge in *F*. *graminearum*, but the effect of weather conditions was not thoroughly investigated.

With a continuous source of inoculum in the current study, ascospores were discharged from perithecia on most days (i.e., on 70% of the days). This frequency of ascospore trapping was higher than in previous studies in which the inoculum sources were inoculated maize kernels or residues distributed on the soil surface of wheat crops [19, 21, 34]. In these previous studies, the inoculum sources developed perithecia under field conditions and therefore likely produced a heterogeneous population of perithecia of different ages, such that the availability of mature ascospores may have been a factor limiting the presence of airborne ascospores on some days.

Ascospore discharge was most affected by rain and vapor pressure deficit, and most of the ascospore discharges (84% of total discharges) occurred on days with R ≥ 0.2 mm or with a VPD ≤ 11 hPa. Similarly, most ascospore discharge peaks (70% of peaks) occurred on days with R ≥ 0.2 mm and VPD ≤ 6.35 hPa. Vapor pressure deficit is the difference between the amount of moisture in the air and the amount of moisture that the air can hold when it is saturated at a given temperature: low VPD indicates closer proximity to the dew point, meaning wet air [49]. In this study, VPD was a better predictor of ascospore discharge than RH or T, indicating that discharge is influenced by the combination of these two factors rather than either factor alone. Consistently with our results, Paulitz [22] observed that spore discharge is triggered by a drop in air temperature and a rise in relative humidity, and Paul et al. [47] found a constant, positive effect of the number of hours of air temperature greater than 15°C and relative humidity >90% on spore production and dissemination. VPD has been frequently used to study the relationship between weather and pathogen epidemiology [50–65] but not for *F*. *graminearum* ascospore discharge until the current study.

*F*. *graminearum* ascospores are forcibly discharged from perithecia, and turgor pressure is required to cause the ascus to stretch to the perithecium ostiole [8] and eject ascospores. Based on our results, the water required to increase ascus turgor [8] can be provided either by rain or by atmospheric humidity. This result is in agreement with the previous studies carried out under controlled conditions [12]. The latter authors found that at 20°C, 1 to 2 h of wetting by rain is necessary for ascospore discharge and that discharge occurred at all RH levels between 40% and 100% even though discharge increased with increasing RH. Discharge at the lowest RH seems to contradict our results. In this work, most of the discharges occurred on days with an average VPD of ≤ 11 hPa, which is lower than the VPD found by Trail et al. [12] (in the latter study, VPD at 20°C and 40% RH = 14.1 hPa). However, on days with an average VPD ≥ 11 hPa (which was unfavorable for ascospore discharge in this work), there were several hours in which VPD was lower (as shown in our hourly data of Fig 4), and these hours may have provided sufficient humidity for the perithecium to absorb water. In addition, our discharge data refer to perithecia on maize residues exposed to natural conditions such that the moisture content of the residues changed with the weather conditions [66]. In Trail et al. [12], in contrast, discharge data were obtained with perithecia on carrot agar, which may have provided some water to perithecia even at low RH levels. Ascospore discharge from perithecia on agar blocks declined as concentrations of mannitol and glycerol in the agar increased [12]. It follows that the properties of the medium holding perithecia, and particularly its osmotic pressure, may influence ascospore discharge.

Several field studies have evaluated the influence of rain and other weather variables on numbers of airborne ascospores [7, 19–22, 24, 27, 32, 34, 37, 39, 67–69]. Because of differences in methodology and environmental conditions, the results have been highly variable and sometimes conflicting. As previously mentioned, inconsistent results from the literature could result from researchers simultaneously considering several interrelated processes, all of which depend on periods of favorable weather conditions, which may occur over several days. For instance, when mature perithecia are available at the time of rain, rain may trigger a rapid discharge of ascospores, as occurred in this work. When mature perithecia are unavailable, however, rain may provide the water that moistens crop residues [66], leading to ascospore maturation and a delayed discharge [34].

When a discharge occurred in the current study, the number of *F*. *graminearum* ascospores sampled from the air above perithecia was influenced by VPD and air temperature. Specifically, average temperature was positively correlated with ascospore number on any day, minimum temperature was positively correlated ascospore number on peak days, and maximum temperature was negatively correlated with ascospores numbers on any day or on peak days. Little information is available on how temperature affects discharge of *F*. *graminearum* ascospores. Schmale & Bergstrom [42] reported that discharge in a wind tunnel occurred between 10 and 30°C, with the optimum at 25°C. Manstretta [70] measured patterns of ascospore discharge at 5, 10, 15, 20, 25, 30, 35, and 40°C by enumerating the ascospores ejected by mature perithecia on microscope slides in Petri dishes. Discharge occurred at all temperatures tested: after 24 h, ≤1 ascospore/mm^2^ slide was found at 5, 10, 35, and 40°C; 2 ascospores/mm^2^ were found at 30°C; 80 to 90 ascospores/mm^2^ were found at 15 and 25°C; and approximately 120 ascospores/mm^2^ were found at 20°C. The positive effect of increasing temperatures between 5 and 20°C on ascospore discharge by perithecial fungi has been attributed to the increase in osmotic pressure in the ascus [71]. Reasons for the reduction in ascospore discharge at high temperatures need to be investigated.

Some ascospore discharges (accounting for 2.5% of the total ascospores sampled) occurred on days with no rain and low humidity. Similarly, Trail et al. [12] found that a few ascospores were discharged at 40% RH. The reasons for discharge at low RH levels are unclear. The possibility that these ascospores came from distant sources of *F*. *graminearum* inoculum, i.e., that ascospores traveled substantial distances on air currents [26], cannot be excluded. In agreement with this hypothesis, Osborne & Stein [40] suggested that ascospores are more ubiquitous in the air than previously thought.

In conclusion, this work has provided novel information on the effect of weather conditions on discharge of *F*. *graminearum* ascospores. More specifically, this work has provided: i) simple rules based on rain and vapor pressure deficit for defining days on which ascospores are likely to become airborne (with 84% probability) and days on which peaks of ascospores are unlikely to occur (with 94% probability); and ii) multiple regression equations based on temperature and vapor pressure deficit for predicting numbers of airborne ascospores (with R^2^ > 0.68). This information should be helpful in refining epidemiological models used as decision aids in disease management programs.

To date, several models that predict FHB and/or mycotoxin, expecially deoxynivalenol (DON), in wheat are available and are widely used (see the review of Prandini et al. [72]). In a recent study [73], the performance of two such models (a mechanistic model developed in Italy and an empirical model developed in the Netherlands) were cross-validated. Results for cross-validation of the mechanistic model [74] and empirical model [75] showed that grain contamination was correctly predicted in 93% of the cases. A recently developed mechanistic model for infection by *F*. *graminearum* ascospores considers perithecia production, ascospore maturation and discharge, and finally infection based on published data [76]. Prediction of ascospore discharge in the latter model may be improved by the results of the present work.

## Supporting Information

S1 Table Weather and ascospores trapping data. (PDF)
