# Supplementary material for: Effects of Weather Variables on Ascospore Discharge from Fusarium graminearum Perithecia
Source: PLoS One. 2015 Sep 24;10(9):e0138860. doi: 10.1371/journal.pone.0138860 (PMC4581667; doi:10.1371/journal.pone.0138860)
Supplement: S1 Table — (PDF) [file pone.0138860.s001.pdf]

date	Tav	Tmin	Tmax	Rhav	RHmin	RHmax	Rt	WDt	RH90	VPD	ascospores/m^3
**16/04/2012**	10.5	7.9	14.8	91	77	97	3.8	20	13	1.3	14.5
**17/04/2012**	12.9	8.0	18.3	72	46	95	0.6	1	4	4.7	72.5
**18/04/2012**	10.8	7.2	16.2	81	53	94	0.2	13	8	2.7	13.05
**19/04/2012**	10.1	7.6	13.7	89	74	96	3.4	15	16	1.4	23.2
**20/04/2012**	11.2	8.6	15.9	84	59	97	2.0	12	10	2.4	8.7
**21/04/2012**	13.1	5.1	19.6	68	42	93	0.0	5	3	5.9	4.35
**22/04/2012**	15.1	9.4	19.2	58	29	87	0.0	0	0	7.9	4.35
**23/04/2012**	12.1	8.2	14.9	73	59	86	0.6	3	0	4.0	18.85
**24/04/2012**	13.4	9.7	17.7	69	47	88	3.4	6	0	5.1	42.05
**25/04/2012**	13.4	7.0	20.2	61	36	86	0.0	0	0	7.1	5.8
**26/04/2012**	14.7	6.9	21.2	58	40	78	0.0	0	0	7.9	0
**27/04/2012**	17.5	10.9	24.5	62	41	78	0.0	0	0	8.7	0
**28/04/2012**	19.4	12.5	26.6	67	46	85	0.0	0	0	8.8	0
**29/04/2012**	17.3	14.4	21.4	75	59	92	0.0	0	4	5.3	0
**30/04/2012**	15.2	11.4	17.4	86	75	95	1.8	7	7	2.5	30.45
**01/05/2012**	15.4	13.4	18.4	94	80	97	16.2	20	20	1.2	5.8
**02/05/2012**	15.0	10.3	18.8	79	64	94	0.0	8	8	4.0	27.55
**03/05/2012**	16.7	9.3	23.5	67	44	93	0.0	2	2	7.4	7.25
**04/05/2012**	15.9	9.1	23.0	68	39	91	0.0	4	1	6.8	11.6
**05/05/2012**	16.8	12.2	22.5	67	43	85	0.8	2	0	7.2	2.9
**06/05/2012**	15.4	10.2	21.5	76	52	92	0.2	6	3	4.9	18.85
**07/05/2012**	16.8	10.7	23.5	70	40	91	0.0	3	5	6.9	1.45
**08/05/2012**	16.4	10.1	22.8	74	48	94	0.2	6	5	5.9	5.8
**09/05/2012**	18.7	11.9	24.3	66	48	89	0.0	1	0	8.3	0
**10/05/2012**	21.3	13.9	28.1	63	39	88	0.0	0	0	11.0	0
**11/05/2012**	23.6	16.3	31.4	61	33	83	0.0	0	0	13.5	1.45
**23/04/2013**	13.2	7.8	18.6	77.2	52.4	94.6	0.2	10	8	4.2	18.85
**24/04/2013**	14.1	7.6	20.2	70.6	41.2	93.5	0.0	10	4	5.8	40.6
**25/04/2013**	16.6	10.1	22.5	66.5	46.5	87.8	0.0	0	0	7.2	23.2
**26/04/2013**	16.6	13.9	20.1	76.5	53.7	94.7	0.6	4	3	4.8	36.25
**27/04/2013**	14.6	13.1	15.7	91.2	80.7	97	5.0	18	15	1.5	55.1
**28/04/2013**	15.8	11.7	20	85.1	65.1	97.8	0.2	11	8	3.0	146.45
**29/04/2013**	15.6	13.8	18.3	93.1	84.8	97.3	5.8	10	20	1.3	123.25
**30/04/2013**	17.0	15	19.8	90.9	80.8	98.1	3.4	7	14	1.9	40.6
**01/05/2013**	18.0	13.6	22.3	85.1	67	96.2	0.8	8	11	3.5	68.15
**02/05/2013**	17.7	14.7	22	85.2	71.1	94.4	0.2	2	9	3.2	2.9
**03/05/2013**	17.2	10.8	22.7	67.6	31.9	91.4	0.0	2	2	7.4	4.35
**04/05/2013**	16.9	9	23.3	65.2	48.8	83.5	0.0	0	0	7.6	8.7
**05/05/2013**	15.7	12.6	19.4	82.1	69.3	97.2	18.4	7	6	3.4	4.35
**06/05/2013**	15.2	13.5	18	93.2	82	98.7	2.6	17	18	1.3	87
**07/05/2013**	16.3	12.3	21.9	85.8	66.2	100	0.0	6	12	3.0	7.25
**08/05/2013**	17.4	12.5	23.1	79.9	61.1	94.6	0.0	8	8	4.7	8.7
**09/05/2013**	19.6	13.9	25	73.9	52	93.5	0.0	7	3	6.9	24.65
**10/05/2013**	17.5	15.1	19.5	86.7	74.5	95.7	6.2	10	8	2.8	17.4

**11/05/2013**	18.0	14	23	77.3	54.6	97.4	2.2	10	8	5.4	84.1
**12/05/2013**	17.3	11.9	22.8	67.1	41.3	91.7	0.2	2	3	7.6	107.3
**13/05/2013**	16.3	8.9	23.3	65.1	37.4	93	0.0	6	3	7.9	1.45
**14/05/2013**	17.5	10.8	23.6	64.2	49.1	84	0.0	2	0	8.1	2.9
**15/05/2013**	16.4	10.7	22.2	73.3	52.4	86.3	0.4	9	0	5.5	39.15
**16/05/2013**	14.8	13.5	17.8	93.5	87.5	96.8	31.8	15	22	1.1	131.95
**17/05/2013**	15.9	13.3	20	75.8	46.8	97.6	15.6	9	10	4.9	92.8
**18/05/2013**	14.1	8.6	18.7	77.5	56.5	93.5	4.6	8	5	3.9	31.9
**19/05/2013**	15.6	12.5	19.1	75.0	52.3	96.1	9.6	9	8	4.9	0
**20/05/2013**	15.0	9.5	20.4	66.6	45.7	91.1	0.0	3	2	6.6	0
**21/05/2013**	15.5	9.2	21.6	65.4	43.6	84.9	0.0	0	0	7.0	0
**22/05/2013**	15.6	8.7	21.1	69.3	50.6	87.6	0.0	1	0	6.2	2.9
**23/05/2013**	17.2	12.8	22.3	67.6	49.8	85.2	0.2	3	0	7.0	23.2
**24/05/2013**	9.8	7.2	12.9	86.7	73.7	93.3	42.7	17	12	1.7	65.25
**25/05/2013**	8.7	7.9	9.4	93.0	90.7	96.1	29.3	24	24	0.8	94.25
**26/05/2013**	13.8	7	19.8	59.5	34.6	95.1	0.0	8	5	7.8	11.6
**27/05/2013**	15.6	6.5	22.3	59.8	45.1	77	0.0	2	0	8.1	4.35
**28/05/2013**	15.8	11	19.5	70.2	60.4	86.2	0.0	0	0	5.8	1.45
**29/05/2013**	15.9	12	20	68.4	49.6	92.4	2.2	6	3	6.3	65.25
**30/05/2013**	13.5	9.5	19.5	74.2	50.8	93.4	0.0	1	3	4.6	14.5
**31/05/2013**	15.9	8.5	22.3	63.3	43.2	88.5	0.0	0	0	7.9	4.35
**01/06/2013**	17.6	12.5	22.4	70.8	57.6	86.1	0.0	0	0	6.3	7.25
**02/06/2013**	21.0	14.2	28.3	60.1	36.6	88.7	0.0	2	0	11.9	0
**03/06/2013**	17.6	14.6	22.3	79.2	56.7	93.1	0.0	3	4	4.6	26.1
**04/06/2013**	19.2	12.1	25.6	66.8	42.4	89.4	0.0	7	0	8.9	13.05
**05/06/2013**	19.6	14.6	24.6	69.4	51.3	86.1	0.0	0	0	7.8	1.45
**06/06/2013**	19.5	13.8	25.9	73.3	53.1	90.8	0.0	1	2	7.0	15.95
**07/06/2013**	21.1	13.9	27.9	69.0	46.7	92.1	0.0	0	3	9.3	4.35
**08/06/2013**	21.2	15.7	27.2	69.1	51.5	86.3	0.0	0	0	8.5	1.45
**09/06/2013**	18.6	14.1	22.6	76.5	61.3	93.7	2.6	10	3	5.4	98.6
**10/06/2013**	17.5	12.2	23.5	76.6	55	94.4	0.0	9	7	5.6	208.8
**11/06/2013**	20.9	13.1	28.5	63.6	37.9	86.6	0.0	0	0	10.8	0
**12/06/2013**	22.0	15.2	28.7	63.4	36.6	87.2	0.0	0	0	11.5	7.25
**13/06/2013**	23.4	15.7	30.2	58.3	39.5	73.8	0.0	0	0	13.6	2.9
**14/06/2013**	24.4	18.1	30.5	60.6	44.6	77.9	0.0	0	0	13.4	0
**15/06/2013**	24.1	17.8	31	68.8	51.4	85.8	0.0	0	0	10.6	1.45
**16/06/2013**	24.5	17.2	31	67.9	50.6	89.8	0.0	0	0	11.4	7.25
**17/06/2013**	26.6	19.6	33.3	61.0	42.1	81.5	0.0	0	0	15.4	0
**18/06/2013**	27.8	20.7	34.8	61.8	40.8	79.7	0.0	0	0	16.1	0
**19/06/2013**	27.9	21.2	34.2	62.5	42.1	84.8	0.0	0	0	16.0	0
**20/06/2013**	26.0	21.1	32.5	60.7	45.2	73.6	0.0	0	0	14.4	0
**21/06/2013**	23.5	17	29	57.6	36.9	80.8	0.0	0	0	13.8	0
**22/06/2013**	22.6	15.3	29	59.6	43.3	80.6	0.0	0	0	12.4	0
**23/06/2013**	22.8	15.5	29.3	62.5	42.9	83.6	0.0	0	0	11.8	0
**24/06/2013**	20.3	16.4	24.2	68.3	47.8	86.1	1.8	6	0	8.1	114.55
**25/06/2013**	21.2	14.3	27.6	50.3	27.3	73	0.0	0	0	14.3	0

**26/06/2013**	20.0	15.2	25.2	58.4	41	80.3	0.0	0	0	10.6	8.7
**27/06/2013**	18.2	12.6	25.3	68.8	40	95	13.6	6	5	8.1	8.7
**28/06/2013**	16.3	10.7	23.2	72.1	44	97	0.0	9	7	6.3	33.35
**29/06/2013**	18.6	12	24.2	60.0	43	81	0.0	0	0	9.7	10.15
**30/06/2013**	22.2	13.2	31.2	56.8	33	85	0.0	1	0	14.1	4.35
**01/07/2013**	24.7	16.1	32.5	51.3	33	76	0.0	0	0	17.4	4.35
**02/07/2013**	24.7	15.5	33.2	51.9	32	81.2	0.0	0	0	17.1	2.9
**03/07/2013**	24.1	17.9	30.3	52.3	37	68	0.0	0	0	15.3	0
**04/07/2013**	24.8	17.1	33.2	54.3	34	74	0.0	0	0	16.5	0
**05/07/2013**	27.0	18.6	35.6	50.6	27	74	0.0	0	0	20.9	0
**06/07/2013**	27.1	20.5	34.2	51.6	32	79	0.0	0	0	19.6	2.9
**05/05/2014**	16.7	7.8	25.5	64.7	37	92	0	5	2	8.6	23.2
**06/05/2014**	16.4	8.2	22.9	67	50	93	0	4	2	7.3	53.65
**07/05/2014**	18.3	13.1	24.4	62.3	44	80	0	1	0	8.9	34.8
**08/05/2014**	18	9.8	25.1	67.2	46	92	0	4	2	8.2	1.45
**09/05/2014**	19.7	12.6	27.7	65.4	41	88	0	0	0	9.7	0
**10/05/2014**	21.3	13.3	28.9	61.6	42	84	0	0	0	11.5	0
**11/05/2014**	20	12.6	26.2	61.6	28	92	0	2	2	10.3	0
**12/05/2014**	17.9	8.9	25.6	44	26	73	0	0	0	12.7	0
**13/05/2014**	15.7	9.7	22.5	63.9	37	88	0	4	0	7.6	5.8
**14/05/2014**	17	8.6	25.1	43.9	18	88	0	1	0	13.3	2.9
**15/05/2014**	17.7	9.1	25.6	31.2	17	53	0	0	0	15.4	0
**16/05/2014**	17.6	9.3	25	37.5	22	60	0	0	0	14.1	0
**17/05/2014**	18.5	9.7	27	47.8	23	75	0	0	0	13.2	0
**18/05/2014**	19.3	10.7	26.6	47.7	29	74	0	0	0	13.4	0
**19/05/2014**	17.9	9.7	25.3	56.5	38	72	0	0	0	9.8	0
**20/05/2014**	20	15.5	25.8	57.6	42	71	0	0	0	10.7	2.9
**21/05/2014**	21.7	14.2	28.8	59.5	40	81	0	0	0	12.3	0
**22/05/2014**	20.9	14.6	28.7	69.8	45	92	1.4	4	2	8.9	0
**23/05/2014**	21.5	15.2	28.7	56.9	19	96	0	8	8	13.9	0
**24/05/2014**	20.4	10.4	27.1	49.9	33	78	0	0	0	13.9	0
**25/05/2014**	21.2	13	29.1	46.6	26	66	0	0	0	15.4	0
**26/05/2014**	19	16.6	25.3	67.4	44	89	6	4	0	7.7	1.45
**27/05/2014**	19	13.6	25.6	64.6	42	93	1.6	4	1	8.9	0
**28/05/2014**	19.3	12	25.9	58.8	41	85	0	0	0	10.5	0
**29/05/2014**	18.6	10.8	27	61.6	39	86	0	0	0	9.6	0
**30/05/2014**	20.5	14	28.7	64.4	34	95	3.2	6	4	10.4	0
**31/05/2014**	19.1	13.9	25.9	69.4	40	97	11.2	9	5	7.9	1.45
**01/06/2014**	20.5	12.2	28	53.7	27	87	0	2	0	13.2	0
**02/06/2014**	19.2	13.8	25.6	62.2	41	81	0	0	0	9.5	5.8
**03/06/2014**	19.6	11.8	27.1	64.2	39	89	0.2	7	0	9.9	11.6
**04/06/2014**	20.1	14.2	25.5	65.3	45	87	0	3	0	9.4	8.7
**05/06/2014**	21.9	14.5	28	63.8	45	89	0	0	0	10.9	2.9
**06/06/2014**	22.9	14.8	30.6	56.8	38	79	0	0	0	14.0	5.8
**07/06/2014**	25.3	17.9	32.8	56.2	33	78	0	0	0	16.5	1.45

**08/06/2014**	27.5	19.3	36.3	55.1	32	78	0	0	0	19.7	1.45
**09/06/2014**	28.3	18.9	37.6	57	27	90	0	3	0	20.9	1.45
**10/06/2014**	28.4	19.2	37.3	49.9	25	79	0	0	0	23.4	0
**11/06/2014**	28.9	21.1	37.4	48	23	70	0	0	0	24.1	0
**12/06/2014**	29.2	20.8	37.8	49.3	26	74	0	0	0	24.1	1.45
**13/06/2014**	27.8	21	35.2	50.3	34	67	0.6	1	0	20.6	4.35
**14/06/2014**	23.7	17.1	33.6	69.7	37	96	26.4	10	7	11.7	40.6
**15/06/2014**	20.3	15.8	26.9	76.8	47	97	34.8	9	11	6.8	4.35
**16/06/2014**	21.1	17.1	26.7	64	42	86	0.2	3	0	10.0	1.45
**17/06/2014**	20.6	15.8	26.9	63.4	43	81	0	0	0	9.9	21.75
**18/06/2014**	20.1	14.3	26.2	65.7	43	87	0	0	0	9.3	1.45
**19/06/2014**	22.2	14.8	30	57.9	33	81	0	0	0	13.4	0
**20/06/2014**	24	15.9	31.8	52	23	76	0	0	0	16.5	4.35
**21/06/2014**	24.1	16.8	29.1	61.9	45.5	87	0	2	0	12.8	11.6
**22/06/2014**	22.6	16.2	29.1	69.3	45.5	99.8	0	5	5	10.0	5.8
**23/06/2014**	26.4	20.8	31.9	56.9	41	74	0	0	0	16.2	4.35
**24/06/2014**	25.3	19	32.1	63.3	43	83	0	0	0	13.5	0
**25/06/2014**	21.7	18.7	27.7	77.6	56	89	1	7	0	6.4	18.85
**26/06/2014**	20.2	17	28.4	85.3	52	97	24.6	16	15	4.5	10.15
